# Clinical outcome of femoral neck system versus cannulated compression screws for fixation of femoral neck fracture in younger patients

**DOI:** 10.1186/s13018-021-02517-z

**Published:** 2021-06-09

**Authors:** Huaijian Hu, Jingbo Cheng, Mingli Feng, Zhihua Gao, Jingwei Wu, Shibao Lu

**Affiliations:** Department of Orthopaedics, Xuanwu Hospital Capital Medical University, Changchun Street 45, Xicheng District, Beijing, China

**Keywords:** Femoral neck system, Cannulated screws, Femoral neck fracture, Harris Hip Score, Surgical fixation devices

## Abstract

**Background:**

The clinical outcome of a new fixation device (femoral neck system, FNS) for femoral neck fractures remains unclear. The main purpose of this study was to evaluate two different internal fixation methods for the treatment of femoral neck fractures in patients aged under 60 years.

**Methods:**

We retrospectively studied patients who underwent internal fixation surgery in our hospital for femoral neck fractures between January 2017 and January 2020. Cannulated compression screws (CCS) and FNS groups were divided according to different internal fixation methods. General data (such as sex, age, body mass index, type of fracture) of all patienFemoral neck shorteningts were collected, and joint function was evaluated using the Harris Hip Score (HHS) before and 1 year after surgery. We recorded related surgical complications, including femoral head necrosis, nonunion, and femoral neck shortening.

**Results:**

There were no significant differences in age, sex, or body mass index between the two groups. There was no statistical difference in HHSs between the two groups before surgery. Patients who underwent FNS treatment had longer surgery time (79.75 ± 26.35 min vs. 64.58 ± 18.56 min, *p* = 0.031) and more blood loss (69.45 ± 50.47 mL vs. 23.71 ± 28.13 mL, *p* < 0.001). The degree of femoral neck shortening in the FNS group was significantly lower than that in the CCS group (10.0% vs 37.5%, *p* = 0.036). Regarding postoperative complications, there was no statistical difference in the incidence of femoral head necrosis and fracture nonunion between the two groups.

**Conclusion:**

Patients younger than 60 with femoral neck fractures can obtain satisfactory clinical results with CCS or FNS treatment. FNS has excellent biomechanical properties and shows significantly higher overall construct stability.

## Background

Femoral neck fractures (FNFs) are a common injury in orthopedic practice and result in significant morbidity and mortality [1]. FNFs are most common in the elderly population, but the treatment of FNFs in relatively young patients deserves our attention. For young people, fractures of the femoral neck are usually caused by high-energy trauma, such as falls from high places or high-speed traffic accidents [2]. For young patients, the goal of surgical treatment is to retain the femoral head as much as possible, avoid necrosis of the femoral head, and achieve bone healing. So young patients with FNFs prefer open or closed reduction and internal fixation (CRIF) [2, 3]. Anatomical reduction and effective fixation are essential for obtaining good prognosis and function [4].

For young patients with FNFs, CRIF and open reduction and internal fixation (ORIF) are currently the most widely recognized treatment plans, but there is still a high incidence of postoperative complications, such as avascular necrosis (AVN), fracture nonunion, and femoral neck shortening. A meta-analysis reviewed 1558 FNFs from 41 studies and concluded that the incidence of AVN was 14.3%, and the incidence of nonunion was 9.3% in younger patients [2]. This is accompanied by an enormous socioeconomic burden and medical challenges. Orthopedic surgeons have to choose the most effective implant to treat FNFs, especially in young patients.

For young adults, the treatment of choice is either CRIF or ORIF with cannulated compression screws (CCS) or a dynamic hip screw (DHS) [4]. Among these internal fixations, cancellous lag screws are the most widely used screws in clinical practice. Pauwel type I and most type II fractures may be effectively managed with three parallel cancellous lag screws inserted in an inverted triangular configuration, entering at or above the level of the lesser trochanter. For Pauwel type III, basicervical, and highly comminuted unstable fracture patterns, a DHS offers greater mechanical stability to resist the increased shearing forces generated and should be used in place of cancellous screws [3, 4].

**Fig. 1:**
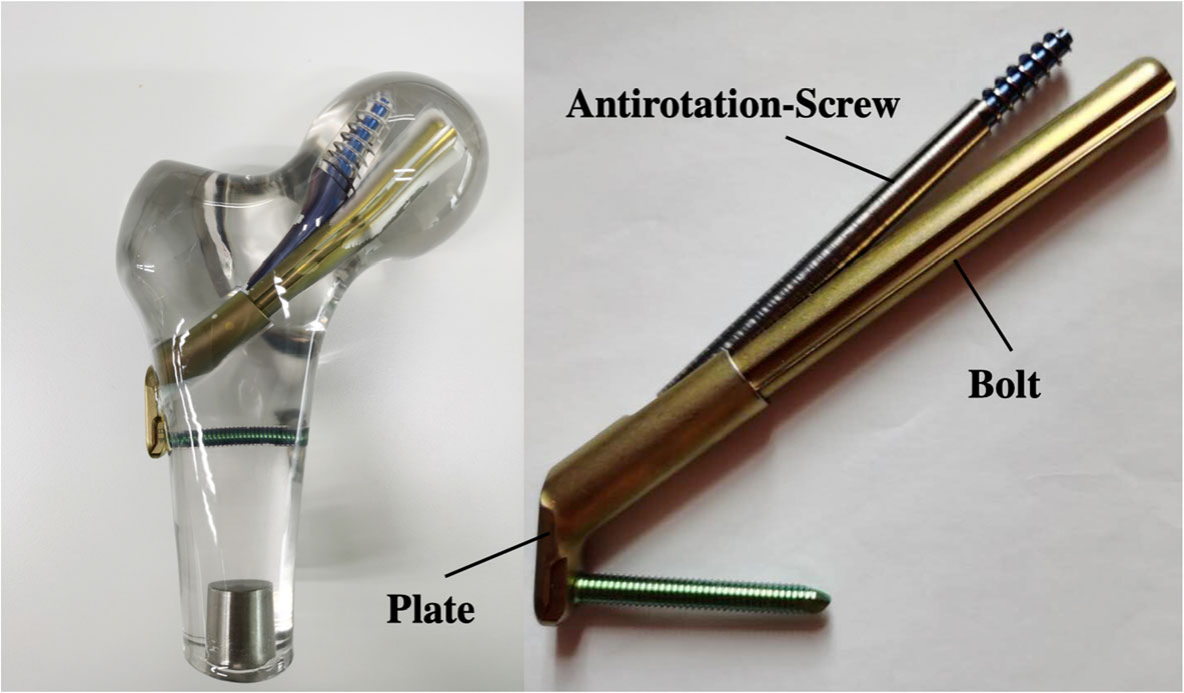
Schematic diagram of femoral neck system

Recently, a new minimally invasive implant has been developed for the dynamic fixation of FNFs called the femoral neck system (FNS) (Fig. 1 showed the schematic diagram of femoral neck system). The implant with its small side plate provides fixation to the femoral shaft while allowing a reduced implant footprint. Fixation of the femoral head is achieved with a screw locked into a bolt to allow these components to slide together along the plate barrel for dynamic fixation.

Currently, there are only biomechanical studies on FNS [5, 6], and there are currently no clinical studies on FNS. The objective of this retrospective comparative study was to evaluate the efficacy and safety of FNS in young patients with FNFs.

## Materials and methods

### Study design

We retrospectively analyzed young patients with FNFs in our hospital between January 2017 and January 2020 (all included patients were less than 60 years old). The demographic and radiological data of these patients were retrospectively collected from the institutional database. The study protocol was reviewed and approved by the institutional review board of the hospital. All patients provided informed consent for participation in the study.

#### Study population

All consecutive younger patients (age<60 years old) with FNFs who were primarily treated with FNS or CCS in our department from January 2017 to December 2019 and with a minimum of 6 months follow-up were included in the study. Patients received CCS treatment from January 2017 to March 2019, and other patients received FNS treatment.

#### Surgical technique

Spinal epidural anaesthesia or general anaesthesia was administered to the patient. All surgeries were performed by the same group of doctors. The patient was placed in the supine position on an orthopedic traction table. After the C-arm X-ray machine confirmed that the fracture was in an adequate reduction position, conventional sterilization was performed.

##### FNS group

**Fig. 2:**
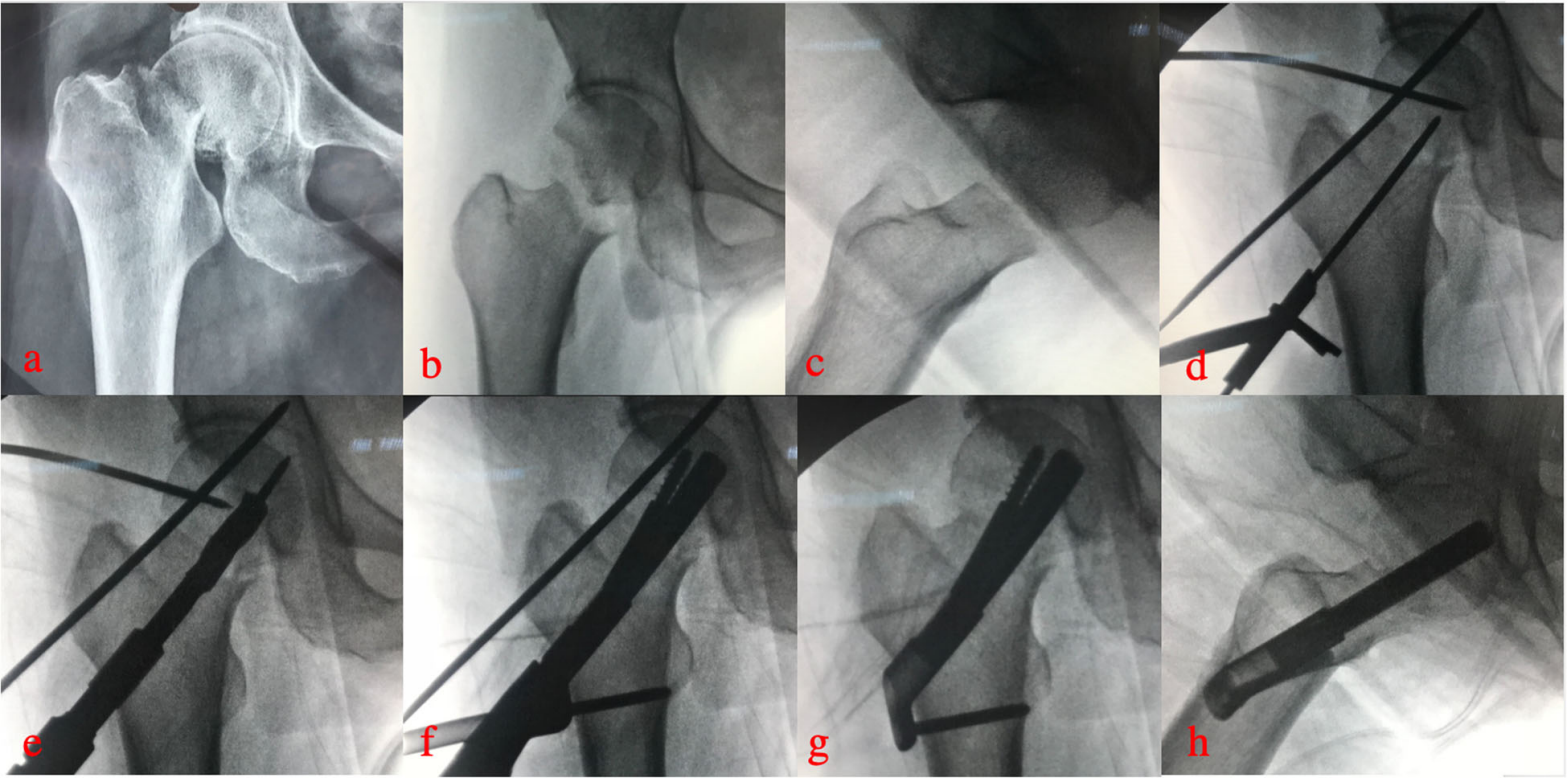
Surgical procedures of treating femoral neck fracture with FNS

The affected limb was slightly abducted and internally rotated. A longitudinal incision of approximately 5cm was made under the greater trochanter. Subsequently, the lateral femoral surface was exposed for satisfactory hardware placement. First, we inserted an anti-rotation wire to fix the fracture. Then, we inserted a second guide wire as the central guide wire using a 130° angled guide. The proper position of the guide wire was confirmed by X-ray. We used a direct measuring device to determine the length and choose the proper implant. We then inserted the implant over the central guide wire into the pre-reamed hole. Next, we drilled a hole for the anti-rotation screw (ARscrew) and inserted it. Interfragmentary compression was applied by turning the insertion screw counterclockwise. The implant position was monitored during compression using X-ray. Finally, we attached a protection sleeve and drilled a hole for the locking screw and inserted it. (Surgical procedures in Fig. 2).

##### CCS group

Three parallel guide pins were inserted into the femoral head along the longitudinal axis of the femoral neck in a triangular configuration under the c-arm perspective. After the guide pins were in the correct position, three cannulated screws were screwed in. Please note that the position of screws should not be lower than the lesser trochanter to reduce the concentration of stress. The distal thread should pass through the fracture line completely. The top of the screw should be 5–10 mm below the femoral head cartilage, and the screw should be as close to the cortex as possible.

### Perioperative management

After ruling out blood disorders and or bleeding tendency preoperatively, low molecular weight heparin sodium (1 mg/kg body weight, once a day) was routinely used for anticoagulation. Antibiotics were administered 0.5 h before the operation. After anesthesia and awakening, the patient was be instructed to actively exercise isometric contraction of the lower extremity muscles, active ankle pump exercises, and active/assisted active hip and knee flexion exercises. Patients with osteoporosis were treated with calcium tablets and diphosphate. Partial weight-bearing training was performed according to the recovery of the affected limb. Approximately 3 months after the operation, walking with a load was permitted according to bone healing. X-ray examination was performed within 3 days after the operation. X-ray follow-up was performed once a month in the first 6 months after surgery and every 6 months thereafter. Hip function assessment was performed 6 and 12 months after the surgery. If the patient had hip pain on the surgical side during follow-up, computed tomography (CT) or magnetic resonance imaging (MRI) of the hip joint was performed to confirm the presence of fracture nonunion or femoral head necrosis.

### Clinical outcome measure

The patients were retrospectively identified from the hospital database. Baseline and follow-up data were acquired from the electronic medical records. Patient records were reviewed and the following data were collected: height, weight, body mass index, time from injury to operation, operation time, blood loss, type of fracture internal fixation, types of fractures (Garden typing and Pauwels classification), and length of clinical follow-up. We used the Mercuriali et al. [7] method to calculate the volume of blood loss. All pre- and postoperative hip radiographs of the study cases were evaluated by the authors who reached a consensual decision for each case regarding the type of FNF (according to Garden and Pauwels classification).

The quality of postoperative fracture reduction was evaluated based on standard anteroposterior and lateral radiographs of the femoral neck of the affected side using the Garden alignment index [8]. Assessment of postoperative fracture healing: There was no obvious percussion pain in the hip joint or lower limbs on the operative side. X-ray or CT showed that the fracture line was blurred, and the original fracture end had continuous cancellous bone trabeculae passing through. Assessment for AVN of the femoral head mainly refers to the standard of Slobogean et al. [9]; that is, if the postoperative X-ray film showed partial collapse of the femoral head or subchondral translucent area. In addition, if the patient had local pain in the hip joint, AVN of the femoral head was suspected, and MRI of the hip joint was performed when indicated. The method of Zlowodzki et al. [10] was used to identify femoral neck shortening.

We used the HHS to evaluate hip joint function preoperatively, and at 6 and 12 months after surgery. At the last follow-up, the Harris Hip scoring system was used to score the function of the hip joint: a full score of 100 points, ≥90 points as excellent, 80–89 points as good, 70–79 points as medium, and <70 points as poor.

### Statistical analysis

The statistical software used for all analyses was SPSS 25.0 (SPSS Inc., Chicago, IL, USA). Continuous variables were reported as mean ± standard deviation (with range). Discrete variables were reported as numbers (percentage of total). Chi-squared tests or Fisher’s exact probability method were used to compare binary variables (demographic data and complication rates).

## Results

This study retrospectively collected data from young patients (<60 years old) with FNFs in our hospital from January 2017 to December 2019 and conducted a follow-up study. All patients received at least 1 year of clinical follow-up. According to different internal fixation methods, they were divided into the FNS and CCS groups. There were no significant differences in age, sex, or body mass index between the two groups (all *p* > 0.05, Table 1). Regarding the classification of FNFs, most patients are classified as Garden type III or type IV.

**Table 1:** Baseline characteristics of all patients with femoral neck fractures treated with FNS or CCS

	FNS	CCS	t/χ2	*P* Value
Cases	20	24		
Gender (male/female)	12/8	14/10	0.013	0.911
Age (year)	50.45 ± 8.45	50.46 ± 9.26	-0.003	0.998
BMI	24.95 ± 2.78	23.61 ± 3.47	1.394	0.171
Garden type				
I	0 (0%)	4 (16.7%)		
II	6 (30.0%)	6 (25.0%)		
III	8 (40.0%)	7 (29.2%)		
IV	6 (30.0%)	7 (29.2%)		
Pauwels type				
I	1 (5.0%)	4 (16.7%)		
II	14 (70.0%)	13 (54.2%)		
III	5 (25.0%)	7 (29.2%)		

Patients who underwent FNS treatment had longer surgery time (79.75 ± 26.35 min vs. 64.58 ± 18.56 mL, *p* = 0.031) and more blood loss (69.45 ± 50.47 mL vs. 23.71 ± 28.13 mL, *p* < 0.001, Table 2).

**Table 2:** Comparison of perioperative characteristics between the FNS and CCS group

	FNS	CCS	t	*P* Value
Operation time (min)	79.75 ± 26.35	64.58 ± 18.56	2.234	**0.031**
Perioperative blood loss (ml)	69.45 ± 50.47	23.71 ± 28.13	3.794	<0.001
Preoperative Harris Score	21.25 ± 4.77	22.63 ± 5.84	−0.843	0.404
Postoperative Harris Score	85.90 ± 5.98	81.92 ± 8.34	1.785	0.081
Excellent	6 (30.0%)	6 (25.0%)		
Good	12 (60.0%)	9 (37.5%)		
Medium	2 (10.0%)	7 (29.2%)		
Poor	0 (0.0%)	2 (8.3%)		
Healing time (months)	3.53 ± 0.90	4.14 ± 1.01	−2.033	**0.050**
Femoral neck shortens (mm)	2.40 ± 1.81	4.54 ± 2.75	−2.979	**0.005**
Garden alignment index				
I	10 (50.0%)	9 (37.5%)		
II	7 (35.0%)	10 (41.7%)		
III	3 (15.0%)	4 (16.7%)		
IV	0 (0.0%)	1 (4.2%)		

There was no statistical difference in HHSs between the two groups before surgery. The postoperative HHSs of the two groups were not significantly different, but the HHSs of the FNS group were slightly higher than those of the CCS group (85.90 ± 5.98 vs. 81.92 ± 8.34, *p* = 0.081, Table 2).

The bone healing time of 3.53 ± 0.90 months in the FNS group was significantly shorter than that of 4.14 ± 1.01 months in the CCS group (*p* = 0.050, Table 2).

Femoral neck shortening occurred in both groups after the surgery. The degree of femoral neck shortening in the FNS group was significantly lower than that in the CCS group (2.40 ± 1.81 mm vs. 4.54 ± 2.75 mm, *p* = 0.005, Table 2).

**Fig. 3:**
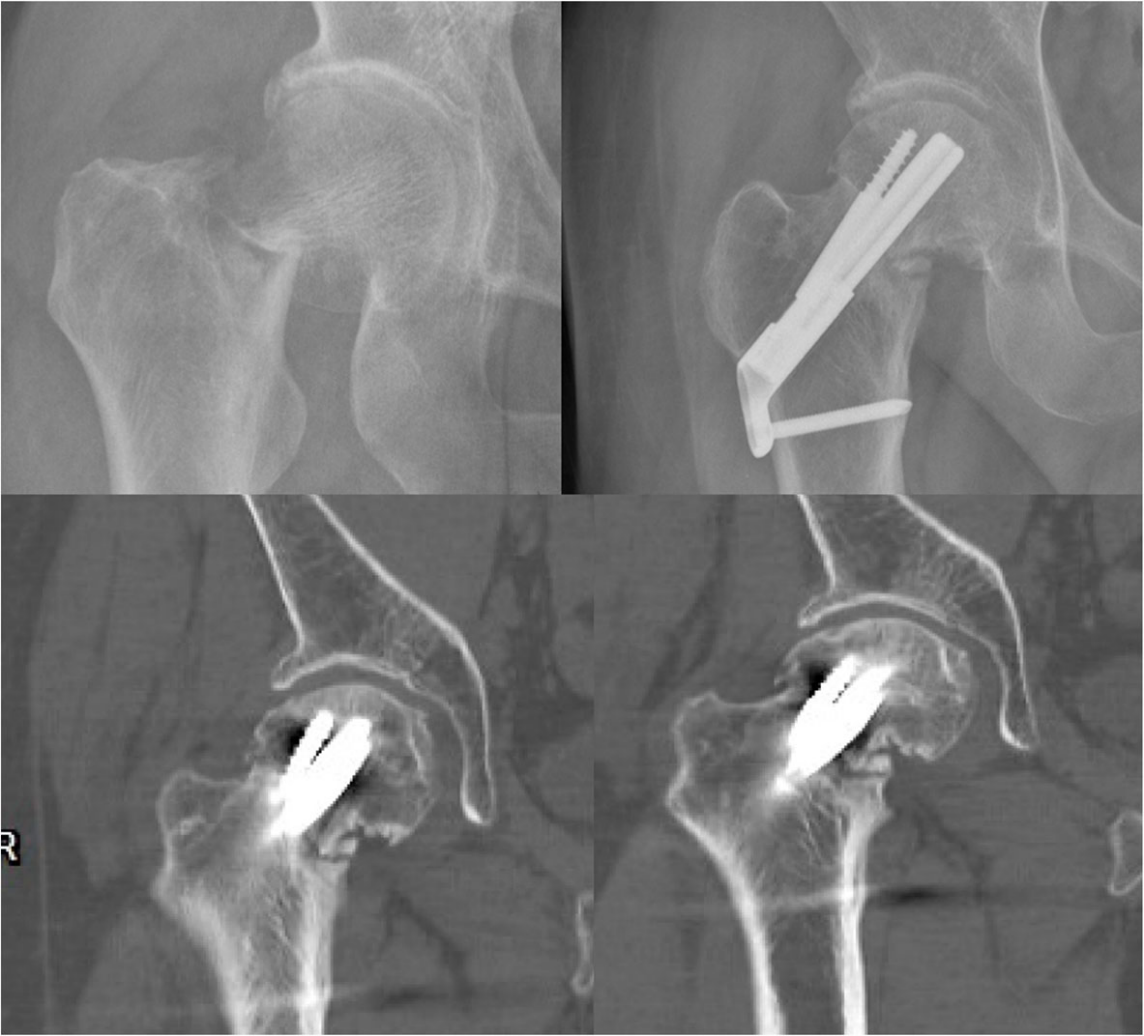
Nonunion of femoral neck fracture (FNS)

**Fig. 4:**
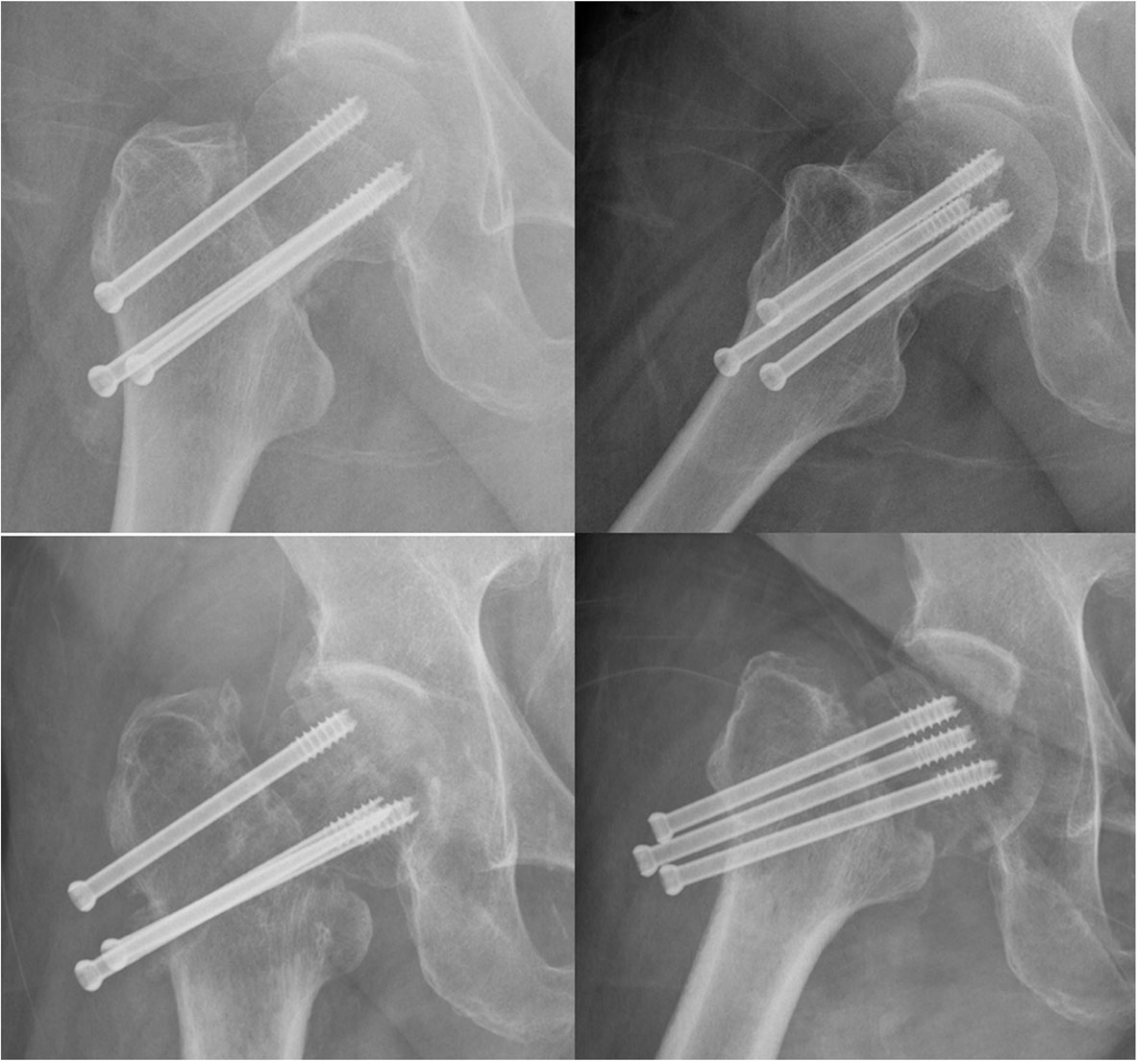
Femoral head necrosis and nonunion of femoral neck fracture (CCS)

Regarding postoperative complications, there was no statistical difference in the incidence of femoral head necrosis and fracture nonunion between the two groups (Table 3) (Figs. 3 and 4). The incidence of femoral neck shortening and screw cut-out in the FNS group was significantly lower than that in the CNS group (10.0% vs. 37.5%, *p* = 0.036; 0.0% vs. 25.0%, *p* = 0.016).

**Table 3:** Comparison of complications between FNS group and CCS group

	FNS	CCS	χ^2^	*P* value
Non-union	2 (10.0%)	3 (12.5%)	0.068	0.795
Femoral head necrosis	1 (5.0%)	3 (12.5%)	0.743	0.389
Femoral neck shortens	2 (10.0%)	9 (37.5%)	4.400	**0.036**
Screw cutout	0 (0.0%)	6 (25.0%)	5.789	**0.016**
Total	3 (15.0%)	12 (50.0%)	5.948	**0.015**

## Discussion

FNFs in patients 60 years of age or younger are challenging injuries to treat because of the high-energy trauma mechanisms and the displaced fracture patterns typically found in this patient population. For young patients with FNFs, fracture reduction and internal fixation are still the most widely accepted treatments, but there is still a high probability of femoral head necrosis, nonunion of fractures, and femoral neck shortening after surgery. Anatomical reduction of fractures and strong and stable internal fixation are key factors in avoiding the aforementioned complications. In the course of fracture management, following the establishment of anatomical reduction, its maintenance is the subsequent logical crucial demand for a fixation device.

### The biomechanical performance of FNS

**Fig. 5:**
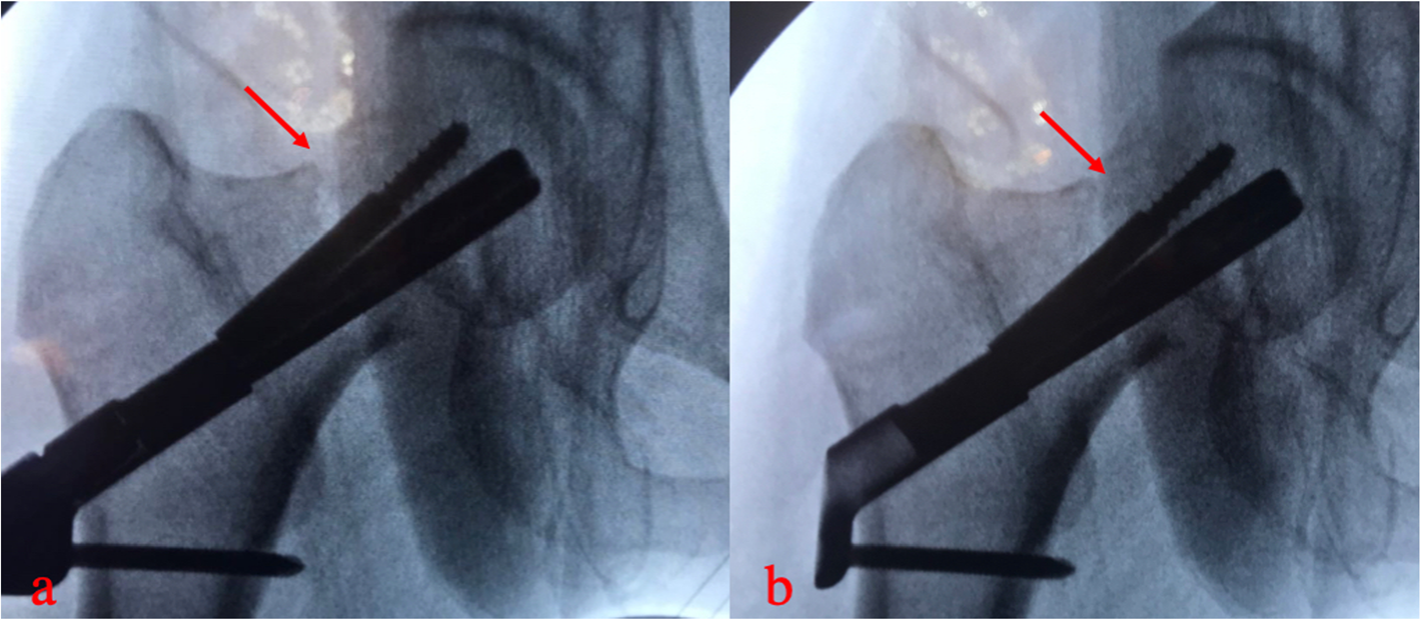
FNS can provide a more strong compression fixation of the fracture site

The recently introduced implant FNS (DePuy Synthes, Zuchwil, Switzerland) (Fig. 1) was developed for the dynamic fixation of FNFs. The FNS includes three parts: an ARScrew, a bolt, and a plate. The plate provides angular stability (a fixed angle between the bolt and the ARScrew). The cylindrical bolt design was intended to maintain reduction during insertion. The bolt also provided angular stability. The integrated bolt and ARScrew provided rotational stability. Stoffel et al. [5] evaluated the biomechanical performance of FNS in comparison with established methods for fixation of FNFs in a cadaveric model. They concluded that the FNS showed significantly higher overall construct stability compared to CCS in an unstable FNF model, and no significant difference between the FNS and the DHS systems was observed with regard to the most clinically relevant parameters. Schopper et al. [6] evaluated the biomechanical performance of the FNS versus Hansson Pin System (Hansson Pins). The study showed that the FNS can be considered as a valid alternative to the Hansson Pin System for the treatment of Pauwels II FNFs by providing superior resistance against varus deformation and performing in a less sensitive way to variations in implant placement. According to previous studies, we may conclude that the FNS can provide similar effects as DHS, achieve strong and stable fixation, and prevent postoperative hip varus. Based on our experience with intraoperative FNS, FNS can provide a more strong compression fixation of the fracture site (Fig. 5). FNS combines the advantages of different existing constructs, such as the minimally invasive insertion technique and retention of more viable bone known for CCS with the increased fracture fixation properties of the DHS system.

### The clinical function and complications of FNS and CCS

In CCS, three cannulated screws apply pressure to the fracture and promote fracture healing. In addition, they occupy a relatively small area in the femoral neck and interfere less with femoral head and neck blood flow. Triangular distribution can form a three-dimensional skeleton and bone tissue, which can decrease the stress of femoral head rotation. It can enhance the intraoperative and postoperative compressive stress between fracture ends, promote close contact between fracture ends, and facilitate fracture healing. However, there is no correlation between the three cannulated screws, and the screw position is easily affected by the subjective and objective factors of the surgeon. Therefore, its ability to resist vertical shear and torsion is poor, which may lead to fracture end loosening and displacement, femoral head necrosis and nonunion, and femoral neck shortening [11, 12]. A biomechanical study showed that FNS and DHS have shown similar results in fracture fixation for parameter cycles to failure and femoral neck shortening [5]. These devices allow for a controlled collapse of the fracture site, leading to an increased stimulus for remodeling. For displaced or unstable fracture patterns, a DHS or FNS offers greater mechanical stability to resist the increased shear forces generated [5, 13]. In our study, there was no statistical difference in the incidence of FNF nonunion (12.5% vs. 10.0%, *p* = 0.795) and femoral head necrosis (12.5% vs. 5.0%, *p* = 0.389) between the CCS and FNS groups. However, the incidence of femoral neck shortening and screw cut-out was significantly higher in the CCS group than in the FNS group (37.5% vs 10.0%, *p* = 0.036). Previous studies have shown that femoral neck shortening after CCS treatment in patients with FNFs may even cause hip dysfunction [14, 15]. Weil et al. [16] showed that the quality of reduction of FNFs had a direct effect on the occurrence of postoperative femoral neck shortening. Osteoporosis can lead to a decrease in fixation grip and resistance to stress at the fracture site, resulting in a decrease in stability and a greater likelihood of femoral neck shortening [17, 18]. In our study, the incidence of femoral neck shortening in the FNS group was significantly lower than that in the CCS group, which may be related to the better mechanical stability and shear resistance of FNS. A previous study also reported that cut-out was a common complication and occurred in 14.5% of patients [19]. The study also implied that a nonparallel and widely spread screw trajectory might interfere with shortening of the osteoporotic femoral neck during fracture healing, leading to the screws possibly cutting out from the femoral head [19]. However, due to the locking mechanism of the plate and screw, there were no patients with screw cut-out in the FNS group. In this study, both the CCS and FNS groups achieved relatively satisfactory functions, and there was no statistically significant difference in postoperative HHSs between the two groups. Our meta-analysis was conducted to analyze the clinical outcomes of two implants (CCS and slide DHS) and concluded that two different types of internal fixation could achieve similar clinical outcomes in terms of the HHS [20]. Factors that affect the clinical outcome after fixation of FNFs primarily depend on the condition of the patients, the degree of fracture displacement, adequacy of internal fixations, and quality of surgical reduction. In our study, the operation time in the FNS group was longer than that in the CCS group, which may be related to the surgical instruments and proficiency. Therefore, it is very important to use the FNS skilfully to shorten the operation time. According to the surgeon’s experience, FNS is significantly better than CCS in applying pressure to the fracture site (Fig. 5).

There are also several limitations in our study: (1) Due to the short clinical application time of FNS, a small number of cases were included in this study; (2) this study only compared the FNS and CCS groups, and the results might be more convincing if the DHS group and the FNS group were compared at the same time.

## Conclusion

In summary, FNS has excellent biomechanical properties and shows significantly higher overall construct stability. Young patients with FNFs can obtain satisfactory clinical results with CCS or FNS treatment. There was no significant difference in the probability of femoral head necrosis and nonunion after surgery. The incidence of femoral neck shortening and screw cut-out in the FNS group was significantly lower than that in the CCS group.

## Data Availability

The datasets analyzed during the current study are available from the corresponding author on reasonable request.

## Abbreviations

FNS: Femoral neck system
CCS: Cannulated compression screw
FNF: Femoral neck fracture
CRIF: Closed reduction and internal fixation
ORIF: Open reduction and internal fixation
AVN: Avascular necrosis
DHS: Dynamic hip screw
CT: Computed tomography
MRI: Magnetic resonance image
HHS: Harris Hip Score
